# Whole-Genome Analysis of *Streptococcus pneumoniae* Serotype 4 Causing Outbreak of Invasive Pneumococcal Disease, Alberta, Canada

**DOI:** 10.3201/eid2707.204403

**Published:** 2021-07

**Authors:** James D. Kellner, Leah J. Ricketson, Walter H.B. Demczuk, Irene Martin, Gregory J. Tyrrell, Otto G. Vanderkooi, Michael R. Mulvey

**Affiliations:** University of Calgary, Calgary, Alberta, Canada (J.D. Kellner, L.J. Ricketson, O.G. Vanderkooi);; Alberta Health Services, Calgary Zone, Calgary (J.D. Kellner, O.G. Vanderkooi);; National Microbiology Laboratory, Public Health Agency of Canada, Winnipeg, Manitoba, Canada (W.H.B. Demczuk, I. Martin, M.R. Mulvey);; University of Alberta, Edmonton, Alberta (G.J. Tyrrell);; Alberta Precision Laboratories–Public Health, Edmonton (G.J. Tyrrell)

**Keywords:** antimicrobial resistance, bacteria, epidemics, homelessness, invasive pneumococcal disease, outbreaks, pneumococcal infections, pneumococcal pneumonia, respiratory infections, streptococci, Streptococcus pneumoniae, Streptococcus pneumoniae serotype 4, vaccines, whole genome sequencing

## Abstract

After the introduction of pneumococcal conjugate vaccines for children, invasive pneumococcal disease caused by *Streptococcus pneumoniae* serotype 4 declined in all ages in Alberta, Canada, but it has reemerged and spread in adults in Calgary, primarily among persons who are experiencing homelessness or who use illicit drugs. We conducted clinical and molecular analyses to examine the cases and isolates. Whole-genome sequencing analysis indicated relatively high genetic variability of serotype 4 isolates. Phylogenetic analysis identified 1 emergent sequence type (ST) 244 lineage primarily associated within Alberta and nationally distributed clades ST205 and ST695. Isolates from 6 subclades of the ST244 lineage clustered regionally, temporally, and by homeless status. In multivariable logistic regression, factors associated with serotype 4 invasive pneumococcal disease were being male, being <65 years of age, experiencing homelessness, having a diagnosis of pneumonia or empyema, or using illicit drugs.

*Streptococcus pneumoniae* causes both invasive and noninvasive disease. Since the introduction of 7-valent and 13-valent protein-polysaccharide conjugated pneumococcal vaccines (PCV7 and PCV13, respectively) for children, vaccine serotype disease has been nearly eliminated among children and reduced indirectly among adults through herd effect (*1**–**4*). PCV7, administered as a 3-dose primary series plus a booster (3+1 dosing schedule) was introduced in Alberta, Canada, in 2002, followed in 2010 by PCV13 (2+1 dosing schedule); both vaccines include serotype 4. In Alberta Province and throughout Canada, invasive pneumococcal disease (IPD) has continued to decline in children <5 years of age since 2010, after PCV13 vaccine introduction, but among older age groups, IPD incidence has remained steady (*5*,*6*). No pediatric cases of IPD caused by *S. pneumoniae* serotype 4 have been diagnosed in Calgary, Alberta, Canada, since 2007 (*3*), although recent data from Calgary showed low levels of serotype 4 carriage in children identified by using PCR but not by using conventional culture (*7*). 

In 2011, IPD caused by *S. pneumoniae* serotype 4 began to increase in adults in the province of Alberta, particularly among persons who were homeless. A previous outbreak in Alberta in 2005–2007 included serotypes 5 and 8, primarily in persons experiencing homelessness and those using illicit drugs (*8*). Homelessness is overrepresented as a factor in adult IPD cases: 18.8% of adults with IPD are homeless, despite only 0.2% of adults in Calgary being homeless (*9*). We conducted this study to examine clinical and demographic factors associated with serotype 4 IPD and to conduct molecular characterization and phylogenetic analysis from whole-genome sequencing (WGS) data on the serotype 4 isolates collected during the outbreak. Our goal was to clarify the dynamics of an outbreak of serotype 4 IPD in a postvaccine community setting where serotype 4 had previously been uncommon. 

## Methods

### Population

**Figure 1:**
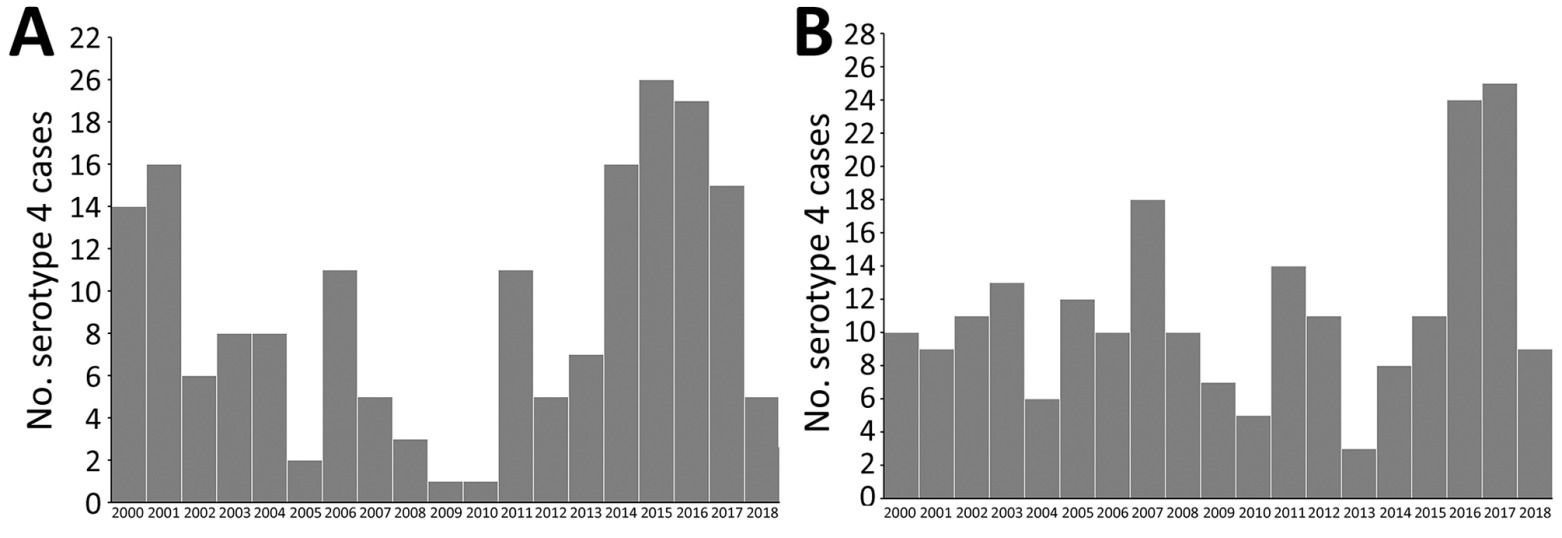
Epidemic curve for *Streptococcus pneumoniae* serotype 4 versus non–serotype 4 causing invasive pneumococcal disease, among adults >18 years of age, Alberta, Canada, 2010–2018. A) Calgary zone of Alberta Health Services; B) capital health zone (Edmonton) of Alberta Health Services.

An inception cohort including all adult case-patients with serotype 4 IPD was identified through population-based surveillance during 2010–2018 in Calgary (2018 population 1,648,385) and Edmonton, Alberta (2018 population 1,393,380). Epidemic curves were generated for Calgary and Edmonton from the number of cases of serotype 4 IPD reported each year during 2000–2018 (Figure 1). We performed WGS to analyze isolates from all patients. We included all adults (≥18 years of age) with IPD reported in the Calgary *S. pneumoniae* Epidemiology Research (CASPER) (*4*) study during 2010–2018 in the clinical analysis.

### Data Collection and Ethics

IPD is a reportable disease to the Ministry of Health in Alberta; therefore, all culture-confirmed cases of serotype 4 IPD in Calgary and Edmonton were identified. All pneumococcal isolates identified by diagnostic microbiology laboratories in Alberta must be submitted to Alberta Precision Laboratories–Public Health for pneumococcal serotyping. Serotyping was performed by quellung reaction (*10*). Clinical information was obtained from chart reviews in Calgary as part of the CASPER study. Ethics approval was provided for the clinical study by the Conjoint Health Research Ethics Board of the University of Calgary. 

### Analysis of Clinical Factors

We collected clinical data on all pneumococcal disease cases in Calgary identified through the CASPER study. Clinical data were not available for cases from Edmonton, so we included only cases from Calgary in the clinical analysis. We used tests of proportions to compare serotype 4 IPD with non–serotype 4 IPD in a univariable analysis to determine clinical and demographic factors and outcomes. We used the Student 2-tailed t-test to compare risk by age as a continuous variable. We chose clinical, demographic, and outcome factors a priori on the basis of biologic plausibility and clinical relevance. For underlying health conditions we sorted patients into 3 groups: those having no underlying conditions increasing risk for IPD; those with underlying conditions but immunocompetent; and those with underlying conditions and immunocompromised, according to Public Health Agency of Canada recommendations for immunization (*11*). For factors with multiple possible responses (e.g., disease manifestation, underlying conditions), which were therefore not possible to collapse into 2 groups, we ran a Fisher χ^2^ test to determine p value. However, although p<0.05 indicates a significant difference between >2 groups, it does not provide information on where the difference occurs.

We used stepwise multivariable logistic regression to analyze clinical and demographic factors and determine adjusted odds ratios and 95% CIs for factors associated with infection from IPD serotype 4 compared with IPD from all other serotypes. We included age as a dichotomous variable: <65 or ≥65 years of age. We did not include indigenous background, intensive care unit (ICU) admission, death, or hospitalization as variables in the model: indigenous status because it is a difficult factor to determine from chart reviews, which are often missing large amounts of data, but its effect was not significant in univariable analysis; ICU admission, death, and hospitalization because they are outcomes and we were interested in clinical factors associated with serotype 4 IPD. We removed smoking status because of nonsignificance and a large amount of missing data.

### Bacterial Identification

We screened isolates using optochin disk susceptibility (Oxoid, http://www.oxoid.com) and tube bile solubility analyses (*12*,*13*). We performed serotyping by quellung reaction using commercial antiserum (Statens Serum Institut, https://en.ssi.dk; SSI Diagnostica, https://www.ssidiagnostica.com) (*10*). We used PCR to test isolates for the presence of the *cpsA* gene if a quellung reaction was not observed (*14*); we verified species identification using *rpoB* (β subunit of RNA polymerase gene) sequence typing (*15*,*16*). 

We determined antimicrobial susceptibilities using Sensititer STP6F (Trek Diagnostics, http://www.trekds.com) broth microdilution panels according to Clinical and Laboratory Standards Institute guidelines (*17*,*18*). We used meningitis resistance breakpoints for nonsusceptibility to penicillin (≥0.12 μg/mL), ceftriaxone and cefotaxime (≥2 μg/mL), and parenteral resistance breakpoints for cefuroxime (≥2 μg/mL). 

### Whole Genome Sequencing Analyses

We conducted WGS analyses on *S. pneumoniae* serotype 4 isolates from Calgary and Edmonton as well as background serotype 4 isolates collected from other provinces in Canada at the National Microbiology Laboratory in Winnipeg, Manitoba, as described elsewhere (*19*). We prepared DNA samples using Epicenter MasterPure Complete DNA and RNA Extraction Kit (Mandel Scientific, https://www.mandel.ca) and created libraries using Nextera sample preparation kits (Illumina, https://www.illumina.com) with 300 bp (n = 140) and 150 bp (n = 50) paired-end indexed reads generated on the Illumina NextSeq platform. We submitted read data for all *S. pneumoniae* serotype 4 isolates from Alberta to the National Center for Biotechnology Information Short Read Archive (BioProject accession no. PRJNA693536). We assessed the quality of the reads using FastQC version 0.11.4 (*20*) and assembled using Shovill (Galaxy version 1.0.4+galaxy0) programs (*21*).

We conducted core single-nucleotide variant (SNV) phylogenetic analysis by using a custom Galaxy version SNVPhyl version 1.0.1b Paired-End (*22*) phylogenomics workflow with minimum coverage = 7, minimum mean mapping quality = 30, and alternative allele ratio = 0.75; we removed highly recombinant regions containing >5 SNVs/500 bp. We visualized phylogenetic trees using FigTree version 1.4.3 (*23*). We determined phylogenetic clades by cluster analysis using ClusterPicker (*24*) with these settings: initial and main support thresholds = 0.9, genetic distance threshold = 0.045, and the large cluster threshold = 10. We used WGS data to identify the presence of macrolide (*ermB*, *ermTR*, *mefA/E*), tetracycline (*tetM*, *tetO*), chloramphenicol (*cat*), trimethoprim/sulfamethoxazole (*folA*, *folP*), penicillin (*pbp1a*, *pbp2b*, *pbp2x*), and fluoroquinolone (*gyrA* S81, *parC* S79/D83/N91) molecular antimicrobial resistance markers (*8**–**12*). We queried virulence factors including *pspA* (pneumococcal surface protein A), *ply* (pneumolysin), *pavA* and *pavB* (pneumococcal adhesion and virulence A and B), *lytA*, *lytB* and *lytC* (autolysins A, B and C), *nanA* and *nanB* (neuraminidase A and B), *rrgA* (pilus-1), *sipA* (pilus-2), *cbpA*, *cbpD*, *cbpG*, and *pce* (choline binding proteins A, D, G and E), *hasA* (hyaluronate lyase), and *zmpC* (immunoglobulin A1 protease) (*13*,*14*). We determined the presence or absence of molecular marker genes in the isolates by querying reference nucleotide sequences against assembled contig files using BLAST (*15*) with the E-value cutoff option set to 10e-100. We used the SNVPhyl workflow program to determine the number of 23S rRNA allele mutations, using an allele of *S. pneumoniae* R6 (locus tag sprr02) as a mapping reference and interrogating the allele counts at nucleotide position 2061 from the resultant variant call files (.vcf). We determined multilocus sequence type (MLST) allelic profiles in silico and queried them using the open-access PubMLST *S. pneumoniae* database (*25*) located at the University of Oxford to determine sequence types (ST).

## Results

### Populations

We identified 190 IPD serotype 4 cases in adults (96 from Calgary, 94 from Edmonton) during 2010–2018 and used WGS to analyze isolates obtained from those patients. A total of 1,008 adults sought treatment at Calgary hospitals with IPD during 2010–2018; of these, 100 (10%) cases involved serotype 4 IPD. For clinical analysis, we completed chart reviews for 97% of the 1,008 IPD cases. For cases without full chart reviews, we collected basic demographic information from notifiable disease reports and laboratory reports and included the information in the analysis when possible. Of the 30 cases without a full chart review, 57% were because of patient refusal to participate in the study. Twelve percent of chart reviews were missing large amounts of information because smoking status was not consistently reported; 56% of reviews lacked clear indication of indigenous status. We included the 967 patients with full information available in the multivariable analysis. 

### Epidemic Curves

In Calgary, after PCV7 introduction, serotype 4 had almost disappeared by 2009–2010. The outbreak among adults peaked in 2015–2016 (Figure 1, panel A). The number of cases of serotype 4 decreased and no cases occurred after July 2018, suggesting resolution of the outbreak. In Edmonton, the decline of serotype 4 after PCV7 introduction was less pronounced and the outbreak had 2 peaks, a smaller one in 2011 and a larger one in 2016–2017 (Figure 1). Serotype 4, which was originally prevalent, declined in the initial period after PCV7 introduction but then increased in 2011 after PCV13 was introduced.

### Clinical Analysis

Homelessness, illicit drug use, alcohol abuse, and smoking were overrepresented as risk factors among patients with cases of serotype 4 IPD in data from the univariable analysis (Table 1). Persons with underlying conditions who were immunocompromised were underrepresented among those with serotype 4 IPD (Table 1). People with serotype 4 IPD were also younger (mean age 47.0 years, 95% CI 44.2–49.8 years) than those with non–serotype 4 IPD (mean age 58.4 years, 95% CI 57.2–59.5 years) during 2010–2018 (t-test p value <0.001). The most common diagnosis for serotype 4 IPD was bacteremic pneumonia (82%). All serotype 4 IPD cases that occurred during 2010–2018 were susceptible to penicillin, ceftriaxone, and erythromycin. In results from multivariable logistic regression, we found that being male, being <65 years of age, experiencing homelessness, having a diagnosis of pneumonia or empyema, or using illicit drugs were associated with serotype 4 IPD (Table 2). Alcohol abuse was not significantly associated with serotype 4 IPD in the multivariable logistic regression, indicating that the association seen in the univariable analysis was because of confounding by another factor (Table 2).

**Table 1:** Results of univariable tests of proportions of patient demographics and clinical characteristics of *Streptococcus pneumoniae* serotype 4 versus non–serotype 4 causing IPD, Calgary, Alberta, Canada, 2010–2018*

Characteristics	Serotype 4, no. (%)	Non–serotype 4, no. (%)	% Difference (95% CI)		p value
Total cases, n = 990	100	890	NA		NA
Age ≥65 y	7 (7.5)	311 (34.9)	27.9 (22.0–33.8)		<0.001
Homelessness	47 (47.5)	129 (14.8)	32.6 (22.5–42.8)		<0.001
Illicit drug use	47 (58.0)	156 (22.5)	35.6 (24.4–46.8)		<0.001
Alcohol abuse	50 (61.7)	238 (34.2)	27.5 (16.3–38.6)		<0.001
Indigenous	13 (40.6)	105 (29.4)	11.2 (–6.4 to 28.9)		0.1862
Current or former smoker	85 (89.5)	579 (74.1)	15.3 (8.4–22.2)		0.001
Severity					
Death ≤30 d	7 (7.0)	114 (12.9)	5.9 (0.5–11.4)		0.09†
Hospitalization	88 (88.0)	775 (88.1)	0.1 (–6.6 to 6.8)		0.9
ICU admission	29 (29.6)	224 (25.9)	3.7 (–5.8 to 13.2)		0.4
Underlying condition an indication for pneumococcal vaccination‡ (*11*)					<0.001§
No underlying condition	46 (46.5)	279 (31.9)	NA		NA
Underlying condition, but immunocompetent	44 (44.4)	356 (40.7)	NA		NA
Underlying condition, immunocompromised	9 (9.1)	237 (27.4)	NA		NA
Primary diagnosis					0.02§
Bacteremia	1 (1.0)	89 (10.0)	NA		NA
Other IPD	1 (1.0)	38 (4.3)	NA		NA
Bacteremic pneumonia	82 (82.0)	613 (68.9)	NA		NA
Empyema	11 (11.0)	86 (9.7)	NA		NA
Pericarditis	7 (0.79)	1 (1.0)	NA		NA
Meningitis	4 (4.0)	56 (6.3)	NA		NA

*IPD, invasive pneumococcal disease; NA, not applicable †p value >0.05 but 95% CI does not cross 0, therefore significant. ‡Underlying conditions exclude alcohol and drug abuse. §p value from χ^2^ test; overall comparison made between groups but not for each level within groups because of multiple levels.

**Table 2:** Results of multivariable logistic regressions of clinical and demographic factors associated with *Streptococcus pneumoniae* serotype 4 versus non–serotype 4 causing IPD, Calgary, Alberta, Canada, 2010–2018 (n = 967)*

Factors associated with serotype 4†	Odds ratio (95% CI)	p value
Male sex	2.1 (1.2–3.7)	0.007
Age ≥65 y	0.3 (0.1–0.7)	0.003
Homelessness	2.4 (1.4–4.1)	0.001
Illicit drug use	2.3 (1.4–3.8)	0.001
Alcohol abuse	0.9 (0.5–1.5)	0.698
Underlying condition an indication for pneumococcal vaccination (*11*)		
No underlying condition increasing risk for IPD	Referent	Referent
Underlying condition, but immunocompetent	0.9 (0.6–1.5)	0.731
Underlying condition, immunocompromised	0.3 (0.1–0.7)	0.003
Primary diagnosis		
Bacteremia or other invasive condition‡	Referent	Referent
Pneumonia or empyema	3.6 (1.1–12.0)	0.034
Meningitis	2.8 (0.6–13.4)	0.202

*IPD, invasive pneumococcal disease. †Smoking status included in initial model but result was not significant and a large amount of data was missing, so it was removed from final model. ‡For example, pericarditis, peritonitis, abscess, endophthalmitis.

### WGS

We conducted WGS analyses on 96 *S. pneumoniae* serotype 4 isolates from Calgary, 94 from Edmonton, and 37 background serotype 4 isolates from the National Microbiology Laboratory, collected from other provinces in Canada (*19*). Illumina MiSeq sequencing yielded an average 817,775 reads/genome, and average genome coverage was 91X. De novo assembly resulted in an average contig length of 45,875 nt and an N50 length of 85,135 nt.

**Figure 2:**
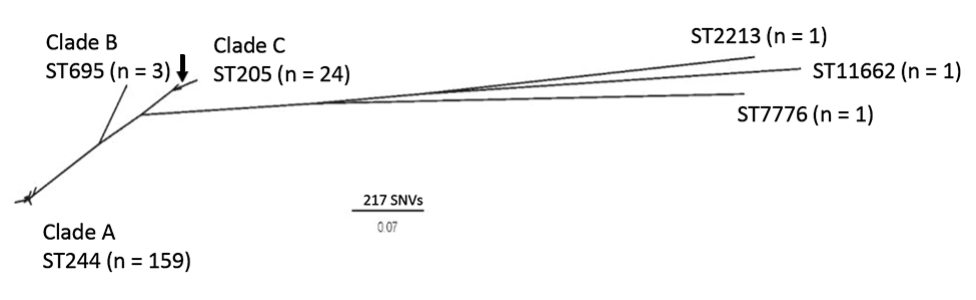
Maximum-likelihood core SNV unrooted phylogenetic tree of 190 *Streptococcus pneumoniae* serotype 4 isolates collected from patients in Alberta, Canada, 2010–2018. A total of 3,097 sites were used in the phylogeny, and 80.7% of the core genome was included. *S. pneumoniae* TIGR4 (arrow; National Center for Biotechnology Information accession number NC_003028.3) was used as a mapping reference. Cluster analysis did not group 1 ST205 isolate and TIGR4 with the other clade C strains. SNV, single nucleotide variant; ST, sequence type.

The 190 *S. pneumoniae* serotype 4 genomes from Alberta clustered into 3 major phylogenetic clades (Figure 2); each clade was associated with an MLST. The largest number 93.7% (n = 159) of isolates were located in clade A and were MLST type ST244. Isolates in clade A were geographically relatively evenly distributed between Calgary (n = 69) and Edmonton (n = 90) and temporally after 2010 (n = 4); ≈19 isolates per year were found during 2011–2019. Clade B included 2 isolates from Edmonton, collected in 2010 and 2012, and 1 from Calgary, collected in 2018, that were ST695, a triple-locus variant of ST244 and ancestor of newly emergent serotype 19A/ST695 clone (*26*). Clade C was associated with ST205 (n = 23) and ST15531 (n = 2), a single-locus variant of ST205. Twenty-three of the 24 isolates in clade C were collected from the Calgary region. Although another ST205 isolate from Edmonton and the TIGR4 reference strain (National Center for Biotechnology Information accession no. NC_003028.3) were proximal to clade C in the phylogenetic tree, ClusterPicker excluded them from the clade based on the clustering thresholds used. A further 2 isolates from Calgary and a third from Edmonton were distant phylogenetic outliers of ST2213, ST7776, and ST11662. Most national background isolates collected from other provinces (n = 36) clustered within the ST205 clade C lineage (n = 23), but the ST244 clade A lineage was predominantly associated with Alberta, with fewer national isolates present (n = 10) (Appendix Figure 1).

**Figure 3:**
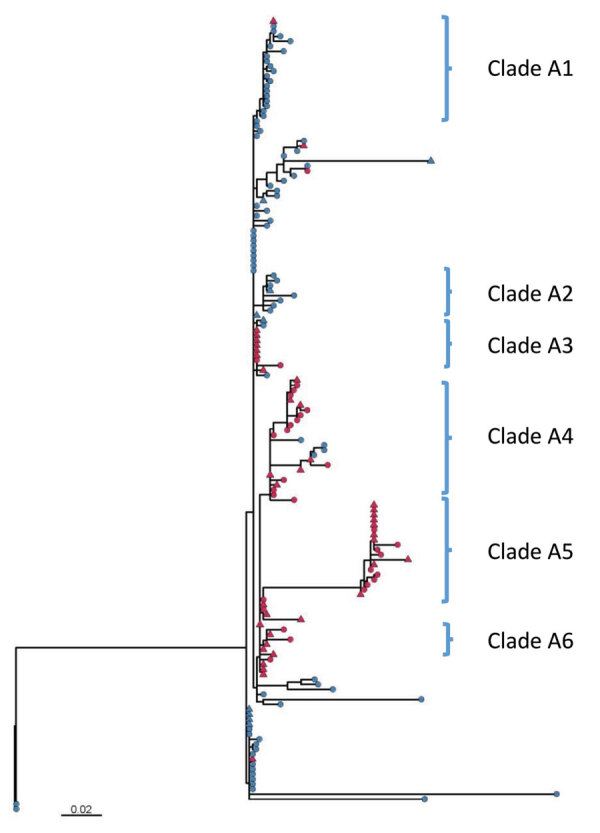
Maximum-likelihood core SNV phylogenetic tree of 159 *Streptococcus pneumoniae* serotype 4 ST244 isolates collected in Alberta, Canada, 2010–2018. A total of 615 sites were used in the phylogeny, and 97.4% of the core genome was included. An internal isolate SC19-3744-P (oldest outlier) was used as a mapping reference and root. Red nodes indicate isolates from the Calgary region, blue nodes those from Edmonton; triangles indicate association with homelessness. Scale bar indicates genetic distance. SNV, single nucleotide variant; ST, sequence type.

Further phylogenetic analysis of the ST244 isolates from Alberta identified 6 major clades with isolates clustered by city (Figure 3). Isolates from Edmonton mainly comprised clades A1 (19 of 21) and A2 (8 of 8), but isolates from the Calgary area comprised clades A3 (9 of 10), A4 (21 of 25), A5 (19 of 19), and A6 (5 of 5). Clade A1 emerged in Edmonton in 2011, and clade A2 followed 4 years later in 2015; in Calgary, clade A6 was first seen in 2011, followed by clades A4 in 2013, and A3 and A5 in 2014, with A5 expanding in 2015 (Appendix Figure 2).

Additional information about homelessness, death, ICU admittance, and risk factors was available for case-patients from Calgary. Clade A1 had the highest proportion of isolates associated with homelessness (7 of 9 isolates), whereas clade A4 had the lowest (7 of 21 isolates; Figure 3). There was also a relatively high number of isolates (3 of 5) in clade A6 and from the miscellaneous nonclustered strains from Calgary (10 of 14) associated with homelessness. Although only about half of the isolates in clade A5 were associated with homelessness (10 of 19), a subgroup of 7 highly related isolates associated with homelessness were identical to each other (no SNV difference). Among the ST205 isolates, 6 of the 23 Calgary isolates were associated with homelessness. We observed no clustering of isolates associated with the other background information (death, risk factors, or ICU admittance). 

### WGS Antimicrobial Susceptibility

Using in silico molecular characterization we found antimicrobial resistance determinants in 7 of the 190 *S. pneumoniae* serotype 4 isolates including single ParC D83 aa substitution (n = 2), *ermB* and *tetM* determinants (n = 1), sole *folA* I100L (n = 2), *folA* I100L, a *folP* amino acid insertion, and *tetM* (n = 1), and a SAMK motif in *pbp2x* and the *folA* I100L and *folP* insert (n = 1). All virulence factors we analyzed were present in the isolates except *cbpA* and *sipA* (pilus-2), which were absent in all strains; *rrgA* (pilus-1) or *zmpC* were absent in the phylogenetic outliers (n = 3).

## Discussion

Outbreaks of IPD have been described most often in vulnerable populations and groups living in crowded conditions, but only a few serotypes have been described in association with outbreaks (*27*). Outbreaks of disease can be characterized as a temporal increase of disease from epidemiologically linked cases. The temporal relation can vary depending on the causative organism. Pneumococcal outbreaks may occur over a period of several years. In this study, phylogenetic analyses were used to support epidemiologic information linking a susceptible population with a particular serotype.

In Calgary we have observed 2 large outbreaks of IPD that particularly affected homeless persons. The one during 2005–2007 was largely caused by serotype 5, although an increase in serotype 8 was also observed (*8*). In the more recent outbreak during 2010–2018, the incidence of serotype 4 increased. Phylogenetic analysis indicated relatively high genetic variability among the serotype 4 isolates collected over this period. Previously, we conducted WGS on a small sample of serotype 5 cases associated with the 2005–2007 outbreak; results indicated that all isolates were from the same genetic clone (*8*). In our analysis of the predominant serotype 4 ST244 clone in Alberta, we observed higher diversity with some clustering regionally in Calgary and Edmonton, as well as some temporal clustering and clustering in homeless persons during 2014–2016. There were also some genetically diverse isolates of *S. pneumoniae* serotype 4 broadly disseminated throughout the community, including among persons who were not homeless. No clustering was observed by age group, gender, site of bacterial isolation, or disease severity, which may indicate that the rise in disease was not because of the emergence of a single, more transmissible clone of serotype 4. Because of the longer temporal period over which pneumococcal outbreaks occur, some degree of genetic drift is expected, with strains being disseminated among the susceptible population and sublineages emerging more acutely in pockets that facilitate transmission, forming diversified subclades within the overall outbreak. The relative diversity among subclades can be thought of as smaller outbreaks of more clonal strains within the overarching dissemination of the original strain.

From a genetic perspective, the phylogeny representing the nationwide breadth of serotype 4 strains had a maximum 1,472 SNVs and an average 192 SNVs between strains (Appendix Figure 1), in contrast with the larger overall outbreak lineage (clade A) with a maximum 148 SNVs and average 22 SNVs. Further clonality can be seen among the subclades with each having ≈5 SNVs difference within and ≈10 SNVs between subclades (Appendix Table). A recent report of an outbreak of serotype 5 IPD in British Columbia, Canada stated that its strains differed by only ≈10 SNVs over a 3.5-year period (*28*).

The rise of serotype 4 IPD cases occurred during a period of widespread PCV13 use in children, raising questions about the reservoirs of this strain. Before that period, during the period of PCV7 use in children, serotype 4 was largely controlled at all ages, reflecting direct immunity in vaccine recipients and indirect immunity in unvaccinated adults. The reemergence of serotype 4 cases, primarily among adults, suggests reduced herd immunity. When Alberta switched from PCV7 to PCV13 in 2010, there was also a switch from a 4-dose schedule of PCV7 to a 3-dose PCV13 schedule for children. This change raises a question about whether the reduced-dose schedule in children, although still providing direct protection, might be less effective in reducing nasopharyngeal carriage, leading to asymptomatic transmission and reduced herd immunity. A study of pneumococcal carriage among children in Calgary has previously shown the near elimination of serotype 4 carriage after the introduction of PCV7, supporting this possible explanation (*29*). More recent studies in children in 2016 and 2018, well after the introduction of PCV13, found that serotype 4 IPD was not identified in any sample tested by conventional culture but was identified in 3.5% of children by using PCR (*7*). It is also possible that PCV13 may not reduce nasopharyngeal carriage and asymptomatic carriage of all vaccine serotypes as effectively as PCV7 did, regardless of the change in number of doses. In support of this possibility, PCV13 has known limited effectiveness to reduce serotype 3 IPD and possibly nasopharyngeal carriage, as described in a 2019 review (*30*).

Serotype 4 IPD was associated with being male or a current user of illicit drugs or experiencing homelessness. Serotype 4 IPD case-patients had lower odds of having an immunocompromising illness, which may be partially associated with being younger, although the association remained when we adjusted for age. We previously reported that IPD is significantly overrepresented in homeless persons compared with the general population, regardless of season (*9*). Although the 23-valent pneumococcal polysaccharide vaccine is recommended for homeless persons in Canada, among those for whom we were able to obtain records, vaccination rates were very low (*9*,*11*).

The main limitation of this study is that we had complete clinical data only from Calgary. In addition, the total population of the surveillance area was 3,041,765 and for a relatively rare disease like IPD, local random variation in prevalent serotypes may limit the generalizability of our results.

Similar to serotype 5 during the 2005–2007 outbreak, serotype 4 also migrated across a large geographic area in western Canada and was seen in Victoria, British Columbia (*31*,*32*). Pneumococcal outbreaks have been reported in overcrowded jails, homeless shelters, and care homes (*8*,*31*,*33**–**36*). One study found recurrent infections were 5-fold higher among persons who were homeless than those who were not (*37*). Another study found most outbreaks of pneumococcal disease occurred in crowded settings (*38*). It is clear that homelessness and drug use are risk factors for illness and should be considered indicators for vaccination. Although we acknowledge the challenge of delivering vaccines to homeless persons, on the basis of these results, we recommended a public health initiative, currently under consideration by public health officials in Alberta, to target the homeless population of Calgary for publicly funded vaccination with both PCV13 and 23-valent pneumococcal polysaccharide vaccine. 

Appendix Additional data from whole genome analysis of *Streptococcus pneumoniae* serotype 4 causing an outbreak of invasive pneumococcal disease in Alberta, Canada.

## References

[1] Pilishvili T, Lexau C, Farley MM, Hadler J, Harrison LH, Bennett NM, et al.; Active Bacterial Core Surveillance/Emerging Infections Program Network. Sustained reductions in invasive pneumococcal disease in the era of conjugate vaccine. J Infect Dis. 2010;201:32–41. 10.1086/648593 19947881

[2] Kaplan SL, Barson WJ, Lin PL, Romero JR, Bradley JS, Tan TQ, et al. Early trends for invasive pneumococcal infections in children after the introduction of the 13-valent pneumococcal conjugate vaccine. Pediatr Infect Dis J. 2013;32:203–7. 10.1097/INF.0b013e318275614b 23558320

[3] Leal J, Vanderkooi OG, Church DL, Macdonald J, Tyrrell GJ, Kellner JD. Eradication of invasive pneumococcal disease due to the seven-valent pneumococcal conjugate vaccine serotypes in Calgary, Alberta. Pediatr Infect Dis J. 2012;31:e169–75. 10.1097/INF.0b013e3182624a40 22673137

[4] Kellner JD, Vanderkooi OG, MacDonald J, Church DL, Tyrrell GJ, Scheifele DW. Changing epidemiology of invasive pneumococcal disease in Canada, 1998-2007: update from the Calgary-area *Streptococcus pneumoniae* research (CASPER) study. Clin Infect Dis. 2009;49:205–12. 10.1086/599827 19508165

[5] Demczuk W, Griffith A, Singh R, Montes K, Sawatzky P, Martin I, et al. National laboratory surveillance of invasive streptococcal disease in Canada—annual summary 2017. Winnipeg, Manitoba (Canada): Public Health Agency of Canada; 2017.

[6] Ricketson LJ, Kellner JD. Invasive pneumococcal disease (IPD) trends 1998 to mid-2019 in Calgary, Canada: an interrupted time series analysis: a CASPER study. International Symposium on Pneumococci and Pneumococcal Disease; 2022 Jun 19–23; Toronto, Ontario, Canada. Abstract 104 [cited 2020 Oct 19]. https://cslide.ctimeetingtech.com/isppd20/attendee/confcal/persons/120

[7] Martin I, Lidder R, Ricketson LJ, Demczuk WH, LeBlanc JJ, Sadarangani M, et al. Molecular identification and serotyping of pneumococcal nasopharyngeal carriage vs culture and quellung serotyping in healthy children: a Calgary *S. pneumoniae* Epidemiology Research (CASPER) study. International Symposium on Pneumococci and Pneumococcal Disease; 2022 Jun 19–23; Toronto, Ontario, Canada. Abstract 348 [cited 2020 Oct 19] https://cslide.ctimeetingtech.com/isppd20/attendee/person/663

[8] Vanderkooi OG, Church DL, MacDonald J, Zucol F, Kellner JD. Community-based outbreaks in vulnerable populations of invasive infections caused by *Streptococcus pneumoniae* serotypes 5 and 8 in Calgary, Canada. PLoS One. 2011;6:e28547. 10.1371/journal.pone.0028547 22216100 PMC3246448

[9] Lemay J-A, Ricketson LJ, Zwicker L, Kellner JD. Homelessness in adults with invasive pneumococcal disease (IPD) in Calgary, Canada. Open Forum Infect Dis. 2019;6:ofz362. 10.1093/ofid/ofz362 31419302 PMC6767971

[10] Austrian R. The quellung reaction, a neglected microbiologic technique. Mt Sinai J Med. 1976;43:699–709. 13297

[11] Public Health Agency of Canada. Pneumococcal vaccine: Canadian immunization guide [cited 2020 Dec 23]. https://www.canada.ca/en/public-health/services/publications/healthy-living/canadian-immunization-guide-part-4-active-vaccines/page-16-pneumococcal-vaccine.html#a3

[12] Facklam R, Washington JA. *Streptococcus* and related catalase-negative Gram-positive cocci. In: Balows A, Hausler WJ, Hermann KL, Isenberg HD, Shadom HJ, editors. Manual of clinical microbiology. Washington: American Society for Microbiology; 1991. p. 238–57.

[13] Spellerberg B, Brandt C. *Streptococcus*. In: Jorgensen JH, Carroll KC, Funke G, et al., editors. Manual of clinical microbiology, 11th ed. Washington: American Society for Microbiology; 2015. p. 383–402.

[14] Pimenta FC, Roundtree A, Soysal A, Bakir M, du Plessis M, Wolter N, et al. Sequential triplex real-time PCR assay for detecting 21 pneumococcal capsular serotypes that account for a high global disease burden. J Clin Microbiol. 2013;51:647–52. 10.1128/JCM.02927-12 23224094 PMC3553924

[15] Drancourt M, Roux V, Fournier PE, Raoult D. rpoB gene sequence-based identification of aerobic Gram-positive cocci of the genera *Streptococcus, Enterococcus, Gemella, Abiotrophia*, and *Granulicatella.* J Clin Microbiol. 2004;42:497–504. 10.1128/JCM.42.2.497-504.2004 14766807 PMC344509

[16] Clinical Laboratory and Standards Institute. Interpretive criteria for identification of bacteria and fungi by DNA target sequencing; approved guideline (MM18-A). Wayne (PA): The Institute; 2008.

[17] Clinical and Laboratory Standards Institute. Methods for dilution antimicrobial susceptibility tests for bacteria that grow aerobically, 8th ed (M07–A8). Wayne (PA): The Institute; 2009.

[18] Clinical Laboratory and Standards Institute. Performance standards for antimicrobial susceptibility testing; 22nd informational supplement (M100–S22). Wayne (PA): The Institute; 2012.

[19] Demczuk WHB, Martin I, Hoang L, Van Caeseele P, Lefebvre B, Horsman G, et al. Phylogenetic analysis of emergent *Streptococcus pneumoniae* serotype 22F causing invasive pneumococcal disease using whole genome sequencing. PLoS One. 2017;12:e0178040. 10.1371/journal.pone.0178040 28531208 PMC5439729

[20] Andrews S. FastQC: a quality control tool for high throughput sequence data, version 0.11.4 [cited 2020 June 29] https://www.bioinformatics.babraham.ac.uk/projects/fastqc

[21] Seemann T. Shovill: faster SPAdes assembly of Illumina reads. 2017. [cited 2020 June 21] https://github.com/tseemann/shovill

[22] Petkau A, Mabon P, Sieffert C, Knox NC, Cabral J, Iskander M, et al. SNVPhyl: a single nucleotide variant phylogenomics pipeline for microbial genomic epidemiology. Microb Genom. 2017;3:e000116. 10.1099/mgen.0.000116 29026651 PMC5628696

[23] Rambaut A. FigTree: molecular evolution, phylogenetics and epidemiology, version 1.4.3 [cited 2020 June 15] http://tree.bio.ed.ac.uk/software/figtree

[24] Ragonnet-Cronin M, Hodcroft E, Hué S, Fearnhill E, Delpech V, Brown AJ, et al.; UK HIV Drug Resistance Database. Automated analysis of phylogenetic clusters. BMC Bioinformatics. 2013;14:317. 10.1186/1471-2105-14-317 24191891 PMC4228337

[25] Jolley KA, Bray JE, Maiden MCJ. Open-access bacterial population genomics: BIGSdb software, the PubMLST.org website and their applications. Wellcome Open Res. 2018;3:124. 10.12688/wellcomeopenres.14826.1 30345391 PMC6192448

[26] Tyrrell GJ. The changing epidemiology of *Streptococcus pneumoniae* serotype 19A clonal complexes. J Infect Dis. 2011;203:1345–7. 10.1093/infdis/jir056 21398394 PMC3080906

[27] Hausdorff WP, Feikin DR, Klugman KP. Epidemiological differences among pneumococcal serotypes. Lancet Infect Dis. 2005;5:83–93. 10.1016/S1473-3099(05)70083-9 15680778

[28] Miller RR, Langille MG, Montoya V, Crisan A, Stefanovic A, Martin I, et al. Genomic analysis of a serotype 5 *Streptococcus pneumoniae* outbreak in British Columbia, Canada, 2005–2009. Can J Infect Dis Med Microbiol. 2016;2016:5381871. 10.1155/2016/5381871 27366170 PMC4904568

[29] Kellner JD, Scheifele D, Vanderkooi OG, Macdonald J, Church DL, Tyrrell GJ. Effects of routine infant vaccination with the 7-valent pneumococcal conjugate vaccine on nasopharyngeal colonization with *streptococcus pneumoniae* in children in Calgary, Canada. Pediatr Infect Dis J. 2008;27:526–32. 10.1097/INF.0b013e3181658c5c 18458650

[30] Linley E, Bell A, Gritzfeld JF, Borrow R. Should pneumococcal serotype 3 be included in serotype-specific immunoassays? Vaccines (Basel). 2019;7:4. 10.3390/vaccines7010004 30609868 PMC6466091

[31] Tyrrell GJ, Lovgren M, Ibrahim Q, Garg S, Chui L, Boone TJ, et al. Epidemic of invasive pneumococcal disease, western Canada, 2005-2009. Emerg Infect Dis. 2012;18:733–40. 10.3201/eid1805.110235 22515944 PMC3358065

[32] McKee G, Choi A, Madill C, Marriott J, Kibsey P, Hoyano D II. Outbreak of invasive *Streptococcus pneumoniae* among an inner-city population in Victoria, British Columbia, 2016-2017. Can Commun Dis Rep. 2018;44:317–22. 10.14745/ccdr.v44i12a02 31517952 PMC6707416

[33] Hoge CW, Reichler MR, Dominguez EA, Bremer JC, Mastro TD, Hendricks KA, et al. An epidemic of pneumococcal disease in an overcrowded, inadequately ventilated jail. N Engl J Med. 1994;331:643–8. 10.1056/NEJM199409083311004 8052273

[34] DeMaria A Jr, Browne K, Berk SL, Sherwood EJ, McCabe WR. An outbreak of type 1 pneumococcal pneumonia in a men’s shelter. JAMA. 1980;244:1446–9. 10.1001/jama.1980.03310130024022 7420632

[35] Mercat A, Nguyen J, Dautzenberg B. An outbreak of pneumococcal pneumonia in two men’s shelters. Chest. 1991;99:147–51. 10.1378/chest.99.1.147 1984946

[36] Gleich S, Morad Y, Echague R, Miller JR, Kornblum J, Sampson JS, et al. *Streptococcus pneumoniae* serotype 4 outbreak in a home for the aged: report and review of recent outbreaks. Infect Control Hosp Epidemiol. 2000;21:711–7. 10.1086/501717 11089655

[37] Plevneshi A, Svoboda T, Armstrong I, Tyrrell GJ, Miranda A, Green K, et al.; Toronto Invasive Bacterial Diseases Network. Population-based surveillance for invasive pneumococcal disease in homeless adults in Toronto. PLoS One. 2009;4:e7255. 10.1371/journal.pone.0007255 19787070 PMC2749333

[38] Zivich PN, Grabenstein JD, Becker-Dreps SI, Weber DJ. *Streptococcus pneumoniae* outbreaks and implications for transmission and control: a systematic review. Pneumonia (Nathan). 2018;10:11. 10.1186/s41479-018-0055-4 30410854 PMC6217781

