# Supplementary material for: Whole-Genome Analysis of Streptococcus pneumoniae Serotype 4 Causing Outbreak of Invasive Pneumococcal Disease, Alberta, Canada
Source: Emerg Infect Dis. 2021 Jul;27(7):1867–75. doi: 10.3201/eid2707.204403 (PMC8237880; doi:10.3201/eid2707.204403)
Supplement: Appendix — Additional data from whole genome analysis of Streptococcus pneumoniae serotype 4 causing an outbreak of invasive pneumococcal disease in Alberta, Canada. [file 20-4403-Techapp-s1.pdf]

Article DOI: https://doi.org/10.3201/eid2707.204403

Whole Genome Analysis of *Streptococcus pneumoniae* Serotype 4 Causing Outbreak of Invasive Pneumococcal Disease, Alberta, Canada

Appendix

Appendix Table. Number of single nucleotide variants in the core genome within and between the major phylogenomic clades of *Streptococcus pneumoniae* serotype 4 ST244 isolates collected in Alberta (Figure 3)

Clade	No. isolates	Avg. SNVs within clade	Max. SNVs within clade	Avg. SNVs from next clade†	Min. SNVs from next clade†	Max. SNVs from next clade†
A1	21	4	12	12	6	23
A2	8	6	14	9	6	20
A3	10	2	10	15	6	29
A4	25	12	27	44	32	62
A5	19	4	19	38	30	52
A6	5	5	12	15‡	4‡	22‡

*Avg, average; Max, maximum; Min, minimum; SNV, single nucleotide variants
†Number of SNVs compared to the following closest ancestral clade in the phylogeny.
‡Number of SNVs compared to clade A in the phylogeny.

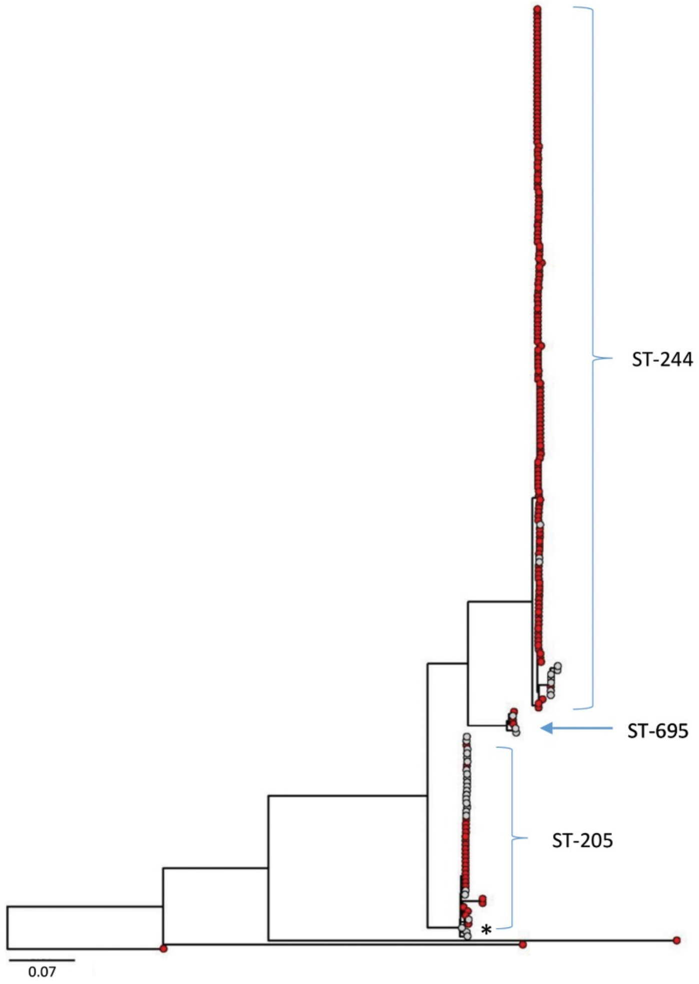

Appendix Figure 1. Maximum likelihood core SNV phylogenetic tree of *S. pneumoniae* serotype 4 isolates collected in Alberta (red nodes, n = 190) during 2010–2018, and other provinces (grey nodes, n = 37). All isolates from other provinces were collected in 2019 except 1 ST205 isolate from 2011 and 1 from 2012. We used a total of 3,075 sites in the phylogeny and included 77.2% of the core genome; we used isolate SC16-4549-P (the oldest outlier isolate) as the tree root and *S. pneumoniae* TIGR4 (NCBI accession number NC_003028.3) as a mapping reference.

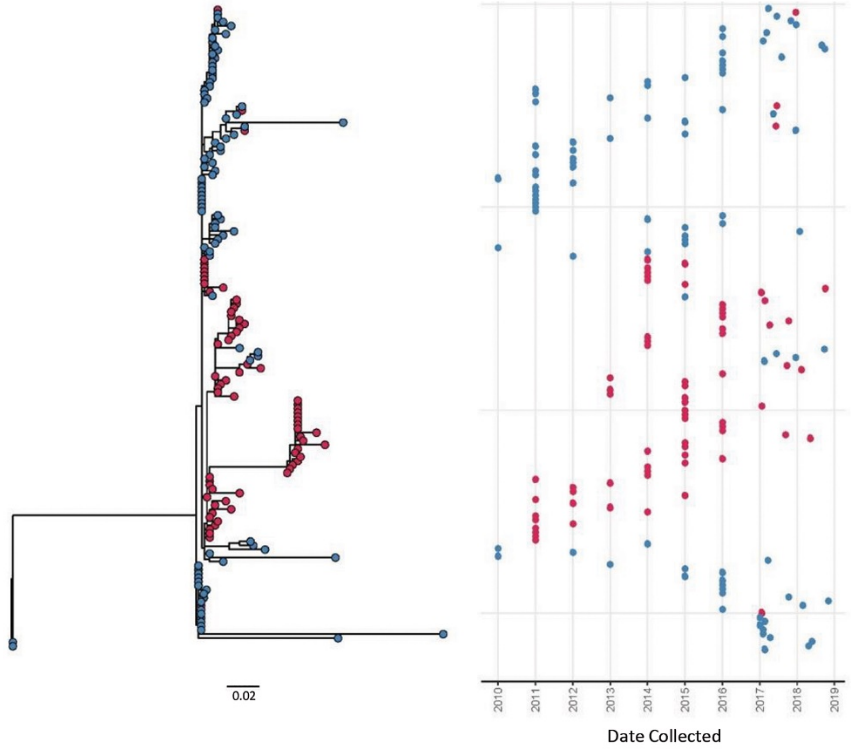

Appendix Figure 2. Temporal distribution of isolates (n=159) collected in Alberta in the maximum likelihood core SNV phylogenetic tree of *S. pneumoniae* serotype 4 ST44 isolates during 2010–2018. We used a total of 615 sites in the phylogeny and 97.4% of the core genome was included. An internal isolate SC19-3744-P (oldest outlier) was used as a mapping reference and root. Isolates from the Calgary region are indicated with red circles, from Edmonton with blue circles.
